# Health Benefits, Costs, and Cost-Effectiveness of Jail-Based Hepatitis C Elimination Strategies

**DOI:** 10.1001/jamainternmed.2026.0190

**Published:** 2026-03-23

**Authors:** Lin Zhu, Lora N. Magaldi, Indrani A. Wagh, Eliza R. Ennis, Marissa B. Reitsma, Danica E. Kuncio, Eman Addish, Sandy R. Varghese, Nathan W. Furukawa, Amanda A. Honeycutt, Yijie Chen, Benjamin P. Linas, Joshua A. Salomon

**Affiliations:** 1 Department of Health Policy, School of Medicine, Stanford University, Stanford, California; 2 Department of Public Health Sciences, University of Miami Miller School of Medicine, Miami, Florida; 3 Philadelphia FIGHT, Philadelphia, Pennsylvania; 4 Philadelphia Department of Public Health, Philadelphia, Pennsylvania; 5 Curran-Fromhold Correctional Facility, Philadelphia Department of Prisons, Philadelphia, Pennsylvania; 6 National Center for HIV, Viral Hepatitis, STD, and TB Prevention, Centers for Disease Control and Prevention, Atlanta, Georgia; 7 National Center for Injury Prevention and Control, Centers for Disease Control and Prevention, Atlanta, Georgia; 8 Section of Infectious Disease, Department of Medicine, Boston Medical Center, Boston, Massachusetts; 9 Department of Epidemiology, Boston University School of Public Health, Boston, Massachusetts

## Abstract

**Importance:**

Injection drug use is a major risk factor for both hepatitis C virus (HCV) infection and incarceration. The high volume and rapid turnover of individuals in jails make this setting a critical access point for hepatitis C elimination among people who inject drugs, with potential downstream benefits for reducing community transmission.

**Objective:**

To evaluate the health benefits, costs, and cost-effectiveness of jail-based HCV interventions.

**Design, Setting, and Participants:**

This cost-effectiveness analysis extended a dynamic network simulation model of HCV transmission through injection equipment sharing among people who inject drugs to incorporate population transitions between jails and communities. Data on justice-involved individuals from the Philadelphia FIGHT program and published literature on people who inject drugs in urban settings in the US were used to model an urban correctional and community setting. Analyses were conducted between April 2024 and February 2026.

**Interventions:**

Jail-based HCV interventions comprising varying combinations of testing at entry, treatment in jail, and HCV navigation services on release.

**Main Outcomes and Measures:**

Changes in person-years of HCV infection, incidence of HCV infection, HCV-related mortality, cumulative quality-adjusted life-years (QALYs), and health care costs across 60 years, and incremental cost-effectiveness ratios (ICERs).

**Results:**

The mean initial age of 1552 simulated people who inject drugs was 32 years. Without jail-based interventions, there were 21 349 person-years of infection, 662 incident infections, and 240 HCV-related deaths per 1000 people who inject drugs over the 60-year time horizon. The combined strategy of testing and treatment plus navigation reduced the cumulative person-years of infection, incidence, and HCV-related deaths in the simulated population by 35% (95% UI, 30%-39%), 47% (95% UI, 41%-54%), and 40% (95% UI, 31%-49%), respectively. The ICER of this strategy was $11 000 per QALY gained compared with no jail-based intervention, well below typical standards for benchmarking cost-effectiveness, which often use thresholds ranging from $50 000 to $150 000 per QALY gained. Providing treatment in jails in addition to testing was cost-saving or yielded lower ICERs compared with providing only testing. Strategies that incorporated navigation yielded lower ICERs than strategies that did not. The results were robust to variations in key parameter values.

**Conclusions and Relevance:**

In this cost-effectiveness analysis using a network simulation model, jail-based HCV elimination interventions, particularly those providing treatment during detention, reduced prevalent and incident HCV cases and HCV-related deaths among people who inject drugs both within and beyond jails. These interventions are a cost-effective strategy for public health decision-makers to consider.

## Key Points

**Question:**

What is the impact of jail-based hepatitis C virus (HCV) interventions on HCV elimination outcomes, and are these interventions cost-effective?

**Findings:**

In this cost-effectiveness analysis using a network simulation model, jail-based HCV interventions reduced HCV incidence and HCV-related deaths among people who inject drugs both within and beyond jail settings by 47% and 40%, respectively, with a cost of $11 000 per quality-adjusted life-year gained compared with the status quo. Additionally, providing treatment in jails yielded greater economic value or cost savings compared with testing alone.

**Meaning:**

Jail-based HCV interventions, particularly those including treatment, substantially improve HCV elimination outcomes in people who inject drugs both within and beyond jail settings and are a cost-effective strategy for public health decision-makers to consider.

## Teaser

This cost-effectiveness analysis examines the impact and value of jail-based hepatitis C interventions among people who inject drugs.

## Introduction

The US has committed to the hepatitis C elimination targets of reducing new hepatitis C virus (HCV) infections by 90% and HCV-related deaths by 65% by 2030.^1^ Progress has been limited with rates of acute hepatitis C steadily increasing from 2011 to 2020 and stabilizing through 2023,^2^ and an estimated 2.2 million people with chronic HCV during 2017 to 2020.^3^ Injection drug use remains the primary transmission route,^2^ targeted strategies for people who inject drugs are therefore crucial to achieving national elimination goals.^4^

Factors including lack of health insurance, housing instability, and criminalization and stigmatization of drug use create barriers to health care access and utilization among people who inject drugs,^5,6^ highlighting the need for innovative strategies for engagement. Substance use disorder prevalence was 5 times higher among justice-involved individuals than the general population,^7,8^ and 25% of people who inject drugs experienced incarceration or detention in the past 12 months,^9^ highlighting carceral facilities as critical venues to reach and engage people who inject drugs in hepatitis C care.

Various HCV-related interventions have been implemented in carceral settings. For example, new hepatitis C care models in California prisons reduced the viremia prevalence by 18%.^10^ The Hepatitis Education Project in Seattle, Washington, implemented a comprehensive viral hepatitis education initiative through both in-person and online education sessions augmented by peer educator training.^11^ Other programs have used telemedicine to provide HCV-related education to correctional health care professionals^12^ or to provide hepatitis C care.^13^

Compared with prisons, jails have higher admission volumes (estimated 7.6 million annual admissions and 664 200 detained at midyear 2023) and shorter average detention periods (32 days),^14^ providing potential for downstream benefits for community-dwelling people who inject drugs. In addition, while prison populations are often dispersed across a state due to centralized placement, jail populations tend to remain localized, creating opportunities for continuity of postrelease care through community navigation by providing support, education, and care coordination after release,^15,16^ as demonstrated by the Philadelphia FIGHT program in Philadelphia, Pennsylvania, which provides HCV testing and treatment services in jails and coordinates postrelease navigation in collaboration with local health care entities and Philadelphia’s jails.^17^

Conversely, the relatively short detention periods and uncertain release dates or transfers can create challenges in having sufficient time for HCV testing and treatment, as well as ensuring care after release. A study in New York City jails demonstrated the feasibility of treating individuals during detention, but also observed high loss to follow-up due to release before treatment completion,^18^ highlighting challenges in health care transitions between jail and community settings. Staff shortages and frequent staff turnover in jails further compound these barriers.^19^ In addition, compared with prisons, which might realize long-term cost savings by treating individuals serving extended sentences, jails face financial disincentives because the transient nature of detention shifts broader public health costs onto already constrained jail budgets, while yielding only minimal long-term savings for the jails themselves.^20^

Nevertheless, the advent of the highly efficacious direct-acting antiviral (DAA) treatments, which require 8 to 12 weeks of oral medication, enhances the feasibility of jail-based HCV interventions, with studies showing high cure rates even with low adherence.^21^ Moreover, jail-based HCV elimination efforts could generate broader public health benefits, despite reallocating societal health care costs to correctional settings.^22^ We therefore evaluated the health benefits, costs, and cost-effectiveness of jail-based HCV elimination strategies among people who inject drugs both within jails and in the community.

## Methods

### Jail-Based HCV Testing, Treatment, and Navigation Strategies

The context of this cost-effectiveness analysis was an urban community setting with medium- to large-sized jails (average daily population >50 persons), similar to that of Philadelphia. The study was determined to be non–human subject research by the Stanford University and Philadelphia Department of Public Health institutional review boards and exempt by the Philadelphia FIGHT institutional review board. This report followed the Recommendations for Conduct, Methodological Practices, and Reporting of Cost-effectiveness Analyses from the Second Panel on Cost-Effectiveness in Health and Medicine (eTable 1 in Supplement 1).^23^ Model simulations were performed using R statistical software, version 3.6 (R Project for Statistical Computing). Analyses were conducted using R statistical software, versions 4.3.2 and 4.5.1 (R Project for Statistical Computing), between April 2024 and February 2026. Parameter inputs for model simulation with corresponding references are displayed in the Table.^24,25,26,27,28,29,30,31,32,33,34,35,36,37,38,39,40,41,42,43,44,45,46,47,48,49,50,51,52,53,54,55,56,57,58,59,60,61,62,63^

**Table.** Parameter Inputs for Model Simulation

Parameter	Value	Source
Jail detention dynamics		
Lifetime detention among PWID, %	86	City of Philadelphia Department of Public Health,^25^ 2018
Annual detention probability, %	43	
Average duration of detention, d	30	Zeng,^26^ 2023
HCV care continuum in jail^a^		
Tested at jail entry, %	91	Trooskin et al,^24^ 2021
DAA initiation in jail among those diagnosed in jail (under jail treatment strategies), %	22	
SVR among those initiated in jail while detained in jail,^a^ %	92	Chan et al,^27^ 2020
Receiving HCV navigation on release, %	48	Trooskin et al,^24^ 2021
HCV care continuum after release^a^		
HCV navigation duration, mo	6	Expert opinion
DAA initiation after release among those diagnosed in jail (status quo), %	16^b^	Hochstatter et al,^28^ 2017; Coyle et al,^29^ 2019; Thompson et al,^30^ 2022; Papaluca et al, ^31^ 2022
DAA initiation after release among those diagnosed in jail (under HCV navigation), %	47	Papaluca et al, ^31^ 2022; Akiyama et al,^32^ 2019
SVR after release (status quo) among those initiated in jail,^a^ %	67^c^	Norton et al,^21^ 2020; Fernández de Cañete Camacho et al,^33^ 2022
SVR after release (under HCV navigation) among those initiated in jail,^a^ %	75	Chan et al,^34^ 2020; Chan et al,^27^ 2020; Morris et al,^35^ 2023
Background HCV care and harm reduction in community^a^		
Monthly HCV testing in community, %	0.61^d^	Bull-Otterson et al,^36^ 2020; Handanagic et al,^37^ 2021
DAA initiation among those diagnosed in community, %	43	Viner et al,^38^ 2015; Younossi,^39^ 2016; Falade-Nwulia,^40^ 2020; Seña et al,^41^ 2016
SVR among those initiated in community, %	90	Alimohammadi et al,^42^ 2018; Graf et al,^43^ 2020
Monthly linkage to SSP, %	4.6	Handanagic et al,^37^ 2021
Monthly linkage to MOUD, %	0.52	Substance Abuse and Mental Health Services Administration,^44^ 2021
Mortality		
SMR for former PWID^e^	1.8	Evans et al,^45^ 2015
SMR for current PWID^e^	6.1	
Additional monthly mortality rate for F4^f^	0.0001	Bruno et al,^46^ 2009
Additional monthly mortality rate for decompensated^f^	0.0128	
Post-SVR mortality multiplier for F4 and decompensated^f^	0.29	van der Meer,^47^ 2012
Health utility (used to calculate QALYs)^g^		
Base (age 14-29 y)	0.928	Hanmer et al,^48^ 2006
Base (age 30-39 y)	0.918	
Base (age 40-49 y)	0.887	
Base (age 50-59 y)	0.861	
Base (age 60-69 y)	0.840	
Base (70-79 y)	0.802	
Base (age 80-100 y)	0.782	
Current PWID	0.68	Pyne et al,^49^ 2008
Former PWID	0.82	
F0-F3, with HCV infection	0.96	van der Meer et al,^47^ 2012; Saeed et al,^50^ 2020
F4, with HCV infection	0.85	
Decompensated, with HCV infection	0.77	
F0-F3, SVR or no infection	1	
F4, SVR or no infection	0.96	
Decompensated, SVR or no infection	0.93	
Costs, $^h^		
Health care, excluding HCV testing and treatment (monthly)		
Current PWID^i^	2165	McCollister et al,^51^ 2018; Murphy et al,^52^ 2019; Lee et al,^53^ 2016
Former PWID^i^	1598	
PWID engaging in MOUD^i^	1155	
F0-F4, active infection^j^	335	Chhatwal et al,^54^ 2013; McAdam-Marx et al,^55^ 2011
F0-F4, SVR/no infection^j^	167	Chhatwal et al,^54^ 2013; McAdam-Marx et al,^55^ 2011; Davis et al,^56^ 2011
Decompensated, active infection^j^	2586	Chhatwal et al,^54^ 2013; McAdam-Marx et al,^55^ 2011
Decompensated, SVR/no infection^j^	1293	Chhatwal et al,^54^ 2013; McAdam-Marx et al,^55^ 2011; Davis et al,^56^ 2011
HCV testing		
Antibody test^k^	14.27	Centers for Medicare & Medicaid Services,^57^ 2025
RNA test^k^	35.09	
Personnel (HCV RNA positive)^l^	77	Schackman et al,^58^ 2018
Personnel (HCV RNA negative)^l^	72	
Treatment initiation^m^		American Association for the Study of Liver Diseases,^59^ 2022
New clinician visit (80% level 4/20% level 3)^n^	135.79	Centers for Medicare & Medicaid Services,^60^ 2025
CBC	6.47	Centers for Medicare & Medicaid Services,^57^ 2025
Liver panel	8.17	
eGFR	5.18	
HBV surface antigen testing	10.33	
HIV antibody/antigen testing	24.08	
DAA treatment (whole course of glecaprevir/pibrentasvir)	19 368.84	Office of Procurement Acquisition and Logistics,^61^ 2025
Treatment completion		American Association for the Study of Liver Diseases,^59^ 2022
RNA test	35.09	Centers for Medicare & Medicaid Services,^57^ 2025
Clinician visit (level 2)	44.48	Centers for Medicare & Medicaid Services,^60^ 2025
Navigation (per person, monthly)	172	Schackman et al,^58^ 2018
Harm reduction interventions (per client, monthly)		
SSP	140	Teshale et al,^62^ 2019
MOUD	592	Fairley et al,^63^ 2021

Abbreviations: CBC, complete blood count; DAA, direct-acting antiviral treatment; eGFR, estimated glomerular filtration rate; F0/1/2/3/4, fibrosis stage 0/1/2/3/4; HBV, hepatitis B virus; HCV, hepatitis C virus; MOUD, medications for opioid use disorder; PWID, people who inject drugs; QALYs, quality-adjusted life-years; SMR, standardized mortality ratio; SSP, syringe services program; SVR, sustained virologic response.

^a^ The testing, treatment initiation, and SVR parameters used for each individual depend on their current status and the intervention received. The decision trees for parameter selection are summarized in eFigure 1 in Supplement 1. Cumulative SVR values were converted into monthly viral clearance probabilities, allowing patients to have different monthly clearance probabilities during treatment depending on their status (eg, detained vs released). Only patients who achieved clearance in both months of the treatment would achieve SVR.

^b^ Due to scarcity of available data, the mean of % DAA initiation estimates from a few sources were used to approximate status-quo % DAA initiation after release, including a study of criminal justice involved adults from Wisconsin, a study of patients in federally qualified health centers in Philadelphia, Centers for Disease Control and Prevention’s report on US Medicaid recipients, and a study of people released from prison in Australia.

^c^ Calculated by adjusting the SVR rate among patients with recent imprisonment (58.33%) for loss to follow-up (excluding patients lost to follow-up increased the rate by a ratio of 1.14 [96.21 divided by 84.04%]).

^d^ The monthly HCV testing rate was 0.64% (7.7% divided by 12) among commercially insured PWID. Ratios of HCV and HIV testing and population sizes of uninsured and insured PWID from the 2018 National HIV Behavioral Surveillance were used to calculate the weighted monthly HCV testing rate among PWID.

^e^ Overall SMRs for all ages. Age-dependent SMRs were generated by fitting a regression to multiple SMR and age group points described in detail in the eAppendix in Supplement 1.

^f^ Calculation is described in detail in the eAppendix in Supplement 1.

^g^ Health utility quantifies an individual’s preference for different health states on a scale from 0 (death) to 1 (perfect health), providing a standardized measure of quality of life. The health utility of an individual in our model was determined by the individual’s age (base utility), injection drug use status, fibrosis stage, and HCV infection status, using a multiplicative model, ie, the overall health-related quality-of-life value of any combination of conditions was estimated by multiplying the utility values of each component condition. Details of the values used are described in the eAppendix in Supplement 1.

^h^ Costs are in 2025 USD.

^i^ The values presented are the means; age-dependent values specified in the eAppendix in Supplement 1 were used. The values were obtained through communication with the authors (details in the eAppendix in Supplement 1).

^j^ Details of calculation are described in the eAppendix in Supplement 1.

^k^ HCV reflex testing was simulated in our study, in which reflex testing describes when people with positive HCV antibody testing results (antibody-positive) are automatically tested for HCV RNA, with antibody-positive results indicating current or previous infection and RNA-positive results indicating current infection.

^l^ The sum of pretest and posttest counseling costs represents personnel cost, and care coordination services represent linkage cost (in Appendix in the cited article); for HCV-positive individuals, the mean of costs of HIV/HCV coinfected and HCV monoinfected patients were used.

^m^ For DAA treatment initiation, components specified in HCV treatment guidance by American Association for the Study of Liver Diseases were included.^59^ We assumed any individual who initiated DAA treatment received a whole course (8 weeks) of glecaprevir/pibrentasvir, which is a simplification of the clinical variation in use of DAA treatments.

^n^ Different levels of clinician visits are associated with different costs. *Current Procedural Terminology* codes 99203 and 99204 were used to identify the costs of level 3 and level 4 clinician visits, respectively.

We evaluated 4 intervention strategies in the main analysis using parameter values derived from published reports from the Philadelphia FIGHT program^24^ (Table): (1) test: HCV testing at jail entry (91% coverage); (2) test plus HCV navigation: testing and 48% receiving postrelease navigation for HCV care; (3) test and treat: testing and 22% receiving treatment initiation in jail; and (4) test and treat plus HCV navigation: a combination of all interventions mentioned in strategies 1, 2, and 3. In addition, we had a reference strategy with no jail-based intervention. HCV navigation was assumed to improve HCV care utilization in the community for a duration of 6 months after release. We incorporated background community interventions, including HCV testing and treatment, syringe services program, and medications for opioid use disorder as a backdrop across all strategies (Table).

### Simulation of Detention

It was assumed that 86% of people who inject drugs would experience jail detention at some point during their lifetime, among whom the annual detention probability was 43%.^25^ The simulations were performed using monthly time steps. Once detained, individuals were assigned a detention period drawn from a Poisson distribution with a mean of 1 month.^26^ Individuals assigned a period of 0 months in the model could still receive jail-based interventions. However, they were classified as released within the same time step, affecting their intervention effectiveness (Table; eFigure 1 in Supplement 1).

### Simulation of HCV Transmission Among People Who Inject Drugs

HCV transmission through the sharing of injection equipment among people who inject drugs in the community was simulated using a previously published dynamic agent-based network model (eTable 2 in Supplement 1).^64,65^ Each simulated individual enters or exits the active injection network by initiation, cessation, relapse of injection drug use, or detention. We assumed that people who inject drugs would reconnect with their previous injection equipment–sharing partner(s) on release if their partner(s) were still injecting. We calibrated the network characteristics and the monthly HCV transmission probability using data from the Social Networks Among Appalachian People study and published literature on injection networks in the US. We compared the prevalences and incidences generated in our model with those reported in systematic reviews for face validity.^64,65,66,67^ We simulated the natural history of HCV infection and fibrosis progression, with mortality dependent on age, injection status, HCV infection status, and liver diseases (eAppendix in Supplement 1).

### Health Utilities and Costs

Health outcomes, including changes in mortality and morbidity, were summarized using quality-adjusted life-years (QALYs). We used age-specific background health utilities based on population norms measured with the EQ-5D.^48^ Additional health decrements associated with HCV infection and other outcomes relating to injection drug use were captured using a multiplicative model (Table).

We included monthly health care costs related to injection status, HCV infection status, and liver conditions for each person (Table). For individuals engaged in syringe services programs, medications for opioid use disorder, or HCV navigation, we also included monthly costs for each service. Costs associated with HCV testing and treatment were recorded by event. We assumed any individual who initiated DAA treatment would receive a whole course of glecaprevir/pibrentasvir for a duration of 8 weeks, which is a simplification of the clinical use of DAA treatments.

### Simulation Setting and Data Analysis

To calculate longer-term outcomes that might be attributed to interventions, we ran intervention scenarios for 10 years and then tracked the further health and economic outcomes for an additional 50 years. After the 10-year intervention period, all intervention scenarios were standardized back to no intervention conditions, and the population was closed to new entrants. For each intervention and sensitivity analysis scenario, we iterated the simulation 500 times. To reduce Monte Carlo errors across comparative runs, we used the same random number seed for different scenarios within each iteration.

We used a health care sector (including carceral health care) perspective for the cost-effectiveness analysis (eTable 1 in Supplement 1) to reflect broader public health outcomes and potential external funding. To evaluate the cost-effectiveness of different strategies relative to a comparison strategy, we calculated the incremental cost-effectiveness ratio (ICER) of each strategy by dividing the incremental costs by the QALYs gained (both averaged over iterations).^68^ Each strategy was compared with the next most effective strategy after eliminating strategies that were dominated (higher costs and lower QALYs) or weakly dominated (higher ICER compared with a more effective strategy). To consider the short-term budgetary impact of the strategies, we computed the incremental costs of testing, treatment, and navigation for jails over a 2-year time horizon. We discounted costs and QALYs at a 3% annual rate.^23^ We calculated point estimates for each outcome as the mean across 500 iterations and quantified stochastic uncertainty by deriving 95% uncertainty intervals (UIs) using the 2.5th and 97.5th percentiles. To characterize uncertainty around the comparative cost-effectiveness of different intervention strategies in relation to stochastic variation in the path-dependent evolution of outcomes within a network of people who inject drugs, we calculated the percentage of the 500 iterations in which each strategy yielded the greatest net benefit (total QALYs gained × willingness-to-pay − total costs) at different willingness-to-pay thresholds.

### Sensitivity Analyses

We conducted sensitivity analyses on key parameters governing (1) intervention coverage in jail; (2) effectiveness of HCV navigation; (3) injection drug use–related mortality; (4) costs of health care services; and (5) health-state disutilities. Given the general lack of evidence to support well-defined measures of uncertainty for most parameters, we conducted 1-way sensitivity analyses based on standard deviations from base-case values, increasing or decreasing the values by 50% compared to the base case. The results of the sensitivity analyses may therefore be interpreted as indicating the degree of change in the outcomes associated with the same relative change in each parameter.^69^ In addition, to test the robustness of outcomes to more unfavorable assumptions about the benefit of HCV navigation on sustained virologic response (SVR) after release, we increased the status-quo SVR rate after release to be equal to that under HCV navigation (from 67% to 75%). Further, to estimate the outcomes in settings that might have sparser injection networks with lower transmission (compared to the urban setting in our base case), we investigated a scenario based on a rural setting of people who inject drugs.^66^ Results in the sensitivity analyses are summarized in terms of how the changes in parameter values alter the ICER for test and treat plus HCV navigation vs no intervention in comparison to the ICER computed in the base-case analysis.

## Results

### Impact of Jail-Based Interventions on HCV Infection Among People Who Inject Drugs

**Figure 1.**
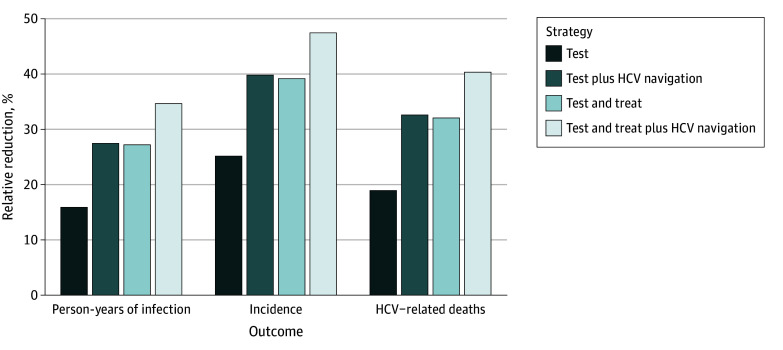
Bar Graph of the Impact of Jail-Based Hepatitis C Virus (HCV) Interventions on HCV Infection Figure 1 presents relative reductions in cumulative person-years of HCV infection, incidence of HCV infection, and hepatitis C–related deaths in the overall population of people who inject drugs during 60 years with 10-year implementation of jail-based HCV testing, treatment, and care navigation interventions compared with no jail-based HCV intervention. The names of the strategies indicate the intervention components in the strategies: test indicates that HCV testing was provided at jail entry; treat indicates that direct-acting antiviral treatments were provided to diagnosed individuals; and HCV navigation on release supports improved direct-acting antiviral treatment initiation and adherence when individuals return to the community. The coverage levels for testing, treatment, and HCV navigation were sourced from published reports from the Philadelphia FIGHT program.

The mean initial age of 1552 simulated people who inject drugs was 32 years. Without jail-based interventions, there were 21 349 person-years of infection, 662 incident infections, and 240 HCV-related deaths per 1000 people who inject drugs over the 60-year time horizon. Figure 1 shows the relative reductions in these HCV outcomes among the overall population of people who inject drugs (within and outside jails) for each strategy. Implementing testing at entry, treatment initiation in jail, and HCV navigation after release together reduced person-years of infection, incidence, and HCV-related deaths by 35% (95% UI, 30%-39%), 47% (95% UI, 41%-54%), and 40% (95% UI, 31%-49%), respectively.

### Short-Term Implications of Jail-Based HCV Interventions

Compared with no intervention, providing testing at entry was estimated to cost an additional $1037 (95% UI, $976-$1106, in 2025 US dollars) per person-year detained during the first 2 years of intervention. Similarly, providing testing and HCV navigation, testing and treatment in jail, and all 3 interventions together were estimated to cost an additional $1435 (95% UI, $1352-$1517), $2468 (95% UI, $1934-$2990), $2531 (95% UI, $1967-$3082) per person-year detained, respectively (eTable 3 in Supplement 1).

### Cost-Effectiveness of Jail-Based HCV Interventions

**Figure 2.**
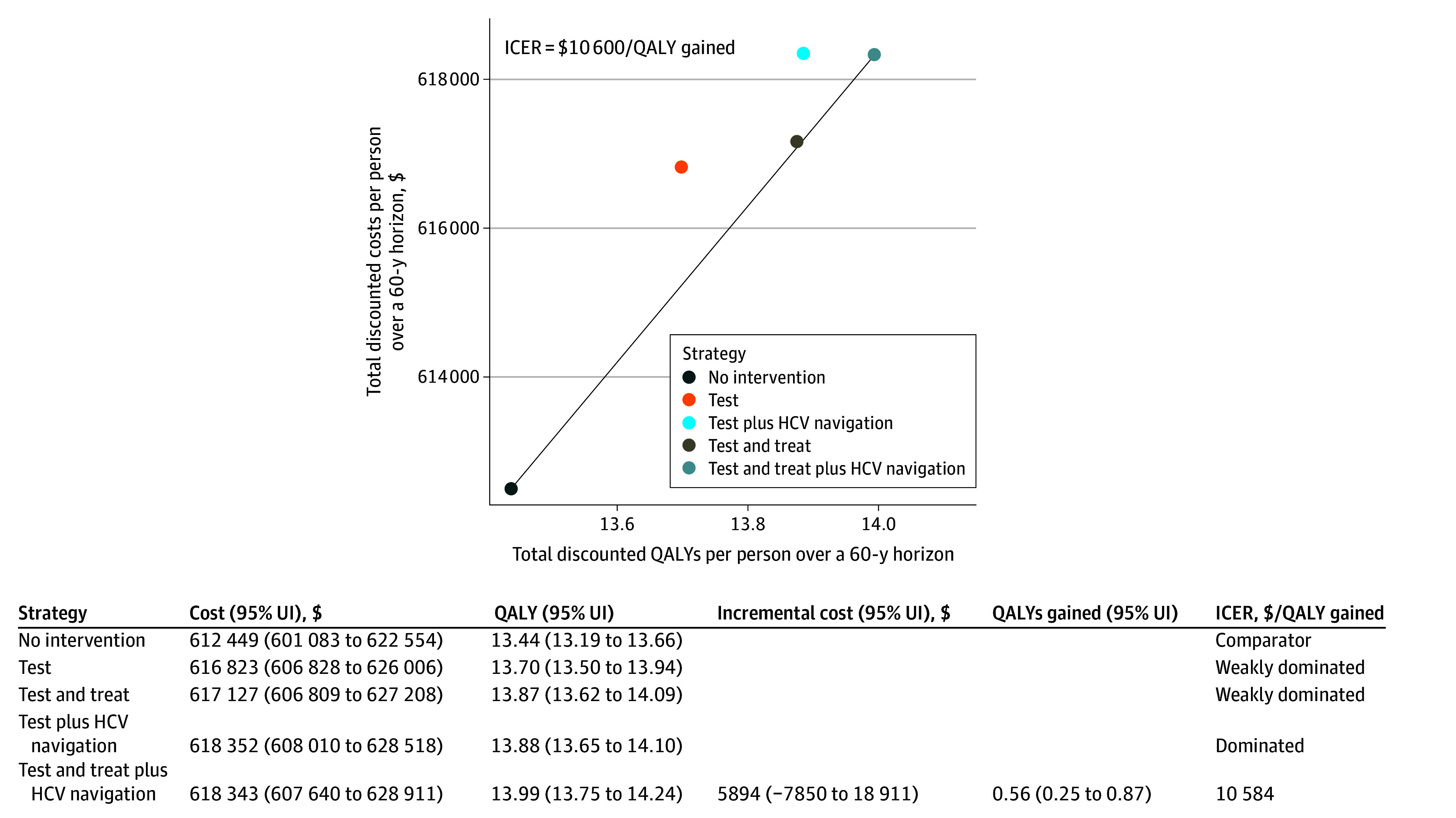
Cost-Effectiveness Plane of Jail-Based Hepatitis C Virus (HCV) Interventions This figure shows the cumulative costs, quality-adjusted life-years (QALYs), and incremental cost-effectiveness ratio (ICER) associated with different HCV testing and treatment strategies in jails from the health care sector perspective shown in 2025 US dollars using a 60-year analytic horizon. The names of the strategies indicate the intervention components in the strategies: test indicates that HCV testing was provided on jail entry, and treat indicates that direct-acting antiviral treatments were provided to diagnosed individuals. The coverages of testing, treatment, and HCV navigation were sourced from published literature (parameter values summarized in the Table). Each point depicts a strategy’s cost and QALY, and the line connects the 2 points left on the cost-effectiveness frontier (excluding dominated and weakly dominated strategies) with its slope indicating ICER. UI indicates uncertainty interval.

Figure 2 shows the cumulative costs, QALYs, and ICERs associated with 10 years of the jail-based HCV interventions over a 60-year time frame in the simulated population of people who inject drugs. Without intervention, the average discounted lifetime costs were $612 400 (95% UI, $601 100-$622 600), and the average QALYs per person were 13.44 (95% UI, 13.19-13.66).

Providing postrelease HCV navigation decreased the ICERs compared with the corresponding strategies without navigation (eg, testing plus navigation yielded a lower ICER than testing alone). Providing treatment in jail was cost-saving or substantially decreased the ICER compared with the corresponding strategies that did not include treatment (eg, test and treat plus navigation cost less than test plus navigation).

After excluding the dominated and weakly dominated strategies, only the most comprehensive strategy remained on the cost-effectiveness frontier, with an ICER of $11 000 per QALY gained compared with no intervention. Although the ICER for the testing and treatment strategy vs no intervention was similar, analysis of stochastic uncertainty indicated that at a willingness-to-pay threshold of $25 000 per QALY gained or higher, the most comprehensive strategy was substantially more likely to provide the greatest net benefit compared to the testing and treatment strategy (eFigure 2 in Supplement 1).

### Sensitivity Analyses

**Figure 3.**
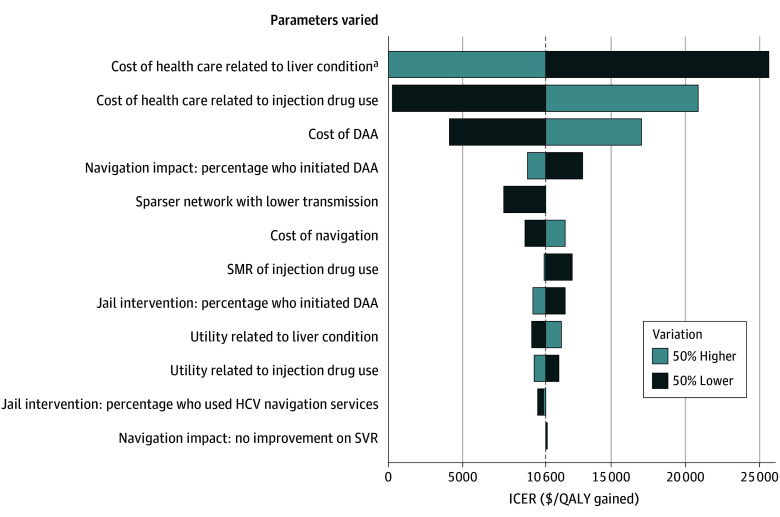
Tornado Graph of the Impact of Variations to Key Parameters on the Cost-Effectiveness of Jail-Based Hepatitis C Virus (HCV) Interventions The figure presents the incremental cost-effectiveness ratios (ICERs) for test and treat plus HCV navigation intervention compared with no intervention when key model parameters were increased or decreased by 50%. Each bar depicts the change in ICER (x-axis) associated with variations in the parameters listed on the y-axis. Light blue bars represent a 50% increase in parameter value, whereas dark blue bars represent a 50% decrease. DAA indicates direct-acting antiviral treatment; QALY, quality-adjusted life-year; SMR, standardized mortality ratio; SVR, sustained virologic response. ^a^Increase in cost of health care related to liver condition by 50% made test and treat plus HCV navigation cost-saving compared with no intervention.

Results of the sensitivity analyses show that while an increase or decrease of 50% in the values of some parameters had a greater impact than others on the ICER of the testing, treatment, and HCV navigation strategy compared with no intervention, none of the changes resulted in an ICER of more than $26 000 per QALY gained (Figure 3). The parameters that had the greatest impact included the cost of health care related to liver conditions, the cost of health care related to injection drug use, and the cost of DAA treatments. This finding is further illustrated in eTable 4 in Supplement 1, which details the contributions of each cost component to the total costs.

## Discussion

Our cost-effectiveness analysis shows that jail-based HCV interventions, even with the base-case coverage (91% tested, 22% initiating treatment, and 48% receiving HCV navigation), could lead to considerable reductions in HCV infection prevalence, incidence, and attributable deaths not only among justice-involved people who inject drugs but within the broader people who inject drugs population, with 10 years of implementation yielding benefits over a 60-year period. The findings suggest that jails might be a highly effective venue for expanding HCV testing and treatment efforts toward elimination. The comparison between strategies also shows that enabling treatment initiation in jail or providing HCV navigation services on release to enhance the continuum of HCV care substantially increased the benefits of the interventions beyond those of testing alone (Figure 1).

The cost-effectiveness analysis indicated that jail-based HCV interventions offer high value for money: requiring an additional $11 000 to gain 1 year of healthy life (Figure 2), which is well below the most conservatively used benchmarks of $50 000 per QALY gained, below which investments are considered cost-effective.^70^ Notably, providing DAA treatment after diagnosis in jail resulted in a substantially more favorable ICER or was even cost-saving compared with not treating during detention. This outcome might result from jails providing a structured environment for people to complete treatment and achieve SVR more effectively than in the postrelease period.^27,33,34,35^ In addition, regardless of whether in-jail treatment is available, providing HCV navigation after release would make the interventions more effective and efficient than those without navigation.

These findings suggest that, despite the restricted budgets of many jails, jail-based HCV treatment might offer substantial economic value at the community level, particularly when integrated into broader public health and local government funding frameworks. Currently, few large jails nationwide have the staffing capacity, technical expertise, and funding necessary to implement HCV testing and treatment programs.^20^ If adequately resourced, such interventions could expand HCV testing and treatment programs to large and possibly medium-sized jails to accelerate hepatitis C elimination progress.

The sensitivity analyses indicate that increases or decreases of 50% in costs, health utilities, intervention coverage, intervention effectiveness, injection-related excess mortality, and HCV transmission levels would not alter the conclusion of the main analysis that jail-based HCV interventions are highly cost-effective. Even with a treatment initiation rate of 11% or an HCV navigation rate of 24%, jail-based treatment and navigation services are still cost-effective compared with no intervention. Among all cost components, background health care costs associated with liver diseases and injection drug use, as well as the costs of DAA treatments, had the greatest impact. We used health care costs related to injection drug use based on published clinical trials, which might be overestimated for community-dwelling people who inject drugs who typically have lower health care access. Our sensitivity analyses (Figure 3; eTable 5 in Supplement 1) indicated that if such costs were lower, the jail-based HCV interventions would be more cost-effective. Although major DAA patents are not set to expire until 2035 in the US, there might be strategies to reduce their costs sooner,^71^ which could also improve the cost-effectiveness of the interventions.

### Limitations

This study has several limitations. First, we did not include the cost of jail coordinating staff or the fixed startup cost due to a lack of published data. However, in our sensitivity analyses increasing intervention costs by 50%, the ICERs remained low. Even at a highly conservative willingness-to-pay threshold of $50 000 per QALY gained, it would take approximately an additional $6000 per person-year detained to make the interventions not cost-effective. Second, we did not conduct a budget impact analysis or cost-effectiveness analysis from the jail perspective due to a lack of data on actual program expenditures and the inherent complexity of economic evaluations in infectious disease interventions. Given the current incentive structure, few jails are positioned to bear these intervention costs for what constitutes a public good. Nevertheless, we emphasize that external investment in jail-based HCV elimination strategies represents a cost-effective approach worthy of consideration by public health decision-makers. Third, studies reported mixed evidence on changes in drug use behaviors^72,73^ and increased drug-related mortality after release.^74^ While higher postrelease mortality could reduce the cost-effectiveness of jail-based interventions, our sensitivity analysis showed that a 50% increase in drug-related SMR had minimal impact. Therefore, we do not expect that elevated postrelease mortality would substantially alter our findings. Fourth, we did not model jail-to-prison transitions, which may occur among a minority of individuals detained in jails. Prisons function as a separate community, and the impact of transitions depends on the level of HCV transmission and availability of care in prisons: lower transmission and adequate care can sustain the intervention benefits, while higher transmission and limited care can decrease the effectiveness and cost-effectiveness of jail-based interventions. Last, we examined the impact of key model inputs in 1-way sensitivity analyses, and did not conduct a probabilistic sensitivity analysis that would jointly propagate second-order uncertainty around model inputs.^69^ However, given the robustness of our primary findings and the relatively low variability in the key model inputs, we believe that it will not change our conclusions.

## Conclusions

In this cost-effectiveness analysis, jail-based HCV testing, treatment, and navigation services on release reduced prevalent and incident cases of hepatitis C and hepatitis C-related deaths among people who inject drugs both within and beyond the jails, while delivering high value for money. Providing treatment in jails yields greater economic value and can even reduce overall costs compared with not treating during detention. The addition of HCV navigation services improves postrelease continuity of care, leading to improved health outcomes at a reasonable cost. Prioritizing investments in impactful and cost-effective strategies like these jail-based interventions represents a promising opportunity to make progress toward achieving hepatitis C elimination.

## Notes

Supplement 1. eTable 1. Reporting Checklist for Cost-effectiveness Analyses eTable 2. Input parameters for simulations using the dynamic network model of people who 11 inject drugs (PWID) eTable 3. Cumulative intervention costs and incremental intervention costs for jails per person-13 year detained over the first 2 years of interventions eTable 4. Contributions of different components to the total cumulative costs over 60 years 15 among different jail-based intervention strategies eTable 5. Cumulative per-person costs, QALYs, and ICERs of jail-based HCV interventions 17 over 60 years with health care costs related to injection drug use reduced by 50% relative to 18 the base-case values eFigure 1. Decision trees of parameter selection on HCV testing, DAA initiation, and DAA 20 completion eFigure 2. Stochastic uncertainty: percentage of simulation iterations in which each strategy 22 had the greatest net benefits eAppendix. Technical Appendix

Supplement 2. Data Sharing Statement
