# Supplementary material for: Health Benefits, Costs, and Cost-Effectiveness of Jail-Based Hepatitis C Elimination Strategies
Source: JAMA Intern Med. 2026 Mar 23;186(5):585–95. doi: 10.1001/jamainternmed.2026.0190 (PMC13010197; doi:10.1001/jamainternmed.2026.0190)
Supplement: Supplement 1. — eTable 1. Reporting Checklist for Cost-effectiveness Analyses eTable 2. Input parameters for simulations using the dynamic network model of people who 11 inject drugs (PWID) eTable 3. Cumulative intervention costs and incremental intervention costs for jails per person-13 year detained over the first 2 years of interventions eTable 4. Contributions of different components to the total cumulative costs over 60 years 15 among different jail-based intervention strategies eTable 5. Cumulative per-person costs, QALYs, and ICERs of jail-based HCV interventions 17 over 60 years with health care costs related to injection drug use reduced by 50% relative to 18 the base-case values eFigure 1. Decision trees of parameter selection on HCV testing, DAA initiation, and DAA 20 completion eFigure 2. Stochastic uncertainty: percentage of simulation iterations in which each strategy 22 had the greatest net benefits eAppendix. Technical Appendix [file jamainternmed-e260190-s001.pdf]

# Supplemental Online Content

Zhu L, Magaldi LN, Wagh IA, et al. Health Benefits, Costs, and Cost-Effectiveness of Jail-Based Hepatitis C Elimination Strategies. *JAMA Intern Med.* Published online March 23, 2026. doi:10.1001/jamainternmed.2026.0190

**eTable 1.** Reporting Checklist for Cost-effectiveness Analyses

**eTable 2.** Input parameters for simulations using the dynamic network model of people who 11 inject drugs (PWID)

**eTable 3.** Cumulative intervention costs and incremental intervention costs for jails per person-13 year detained over the first 2 years of interventions

**eTable 4.** Contributions of different components to the total cumulative costs over 60 years 15 among different jail-based intervention strategies

**eTable 5.** Cumulative per-person costs, QALYs, and ICERs of jail-based HCV interventions 17 over 60 years with health care costs related to injection drug use reduced by 50% relative to 18 the base-case values

**eFigure 1.** Decision trees of parameter selection on HCV testing, DAA initiation, and DAA 20 completion

**eFigure 2.** Stochastic uncertainty: percentage of simulation iterations in which each strategy 22 had the greatest net benefits

**eAppendix.** Technical Appendix

This supplemental material has been provided by the authors to give readers additional information about their work.



© 2026 Zhu L et al. JAMA Internal Medicine.

**eTable 1. Reporting Checklist for Cost-effectiveness Analyses**

| Element | Journal Article | Technical Appendix |
|---|---|---|
| **Introduction** | | |
| Background of the problem | × | |
| **Study Design and Scope** | | |
| Objectives | × | |
| Audience | × | |
| Type of analysis | × | |
| Target populations | × | |
| Description of interventions and comparators (including no intervention, if applicable) | × | |
| Other intervention descriptors (eg, care setting, model of delivery, intensity and timing of intervention) | × | |
| Boundaries of the analysis; defining the scope or comprehensiveness of the study (eg, for a screening program, whether only a subset of many possible strategies are included; for a transmissible condition, the extent to which disease transmission is captured; for interventions with many possible delivery settings, whether only one or more settings are modeled) | × | |
| Time horizon | × | |
| Analytic perspectives (eg, reference case perspectives [health care sector, societal]; other perspectives such as employer or payer) | × | |
| Whether this analysis meets the requirements of the reference case | × | |
| Analysis plan | × | |
| **Methods and Data** | | |
| Trial-based analysis or model-based analysis. If model-based: | × | |
| Description of event pathway or model (describe condition or disease and the health states included) | × | × |
| Diagram of event pathway or model (depicting the sequencing and possible transitions among the health states included) | × | |
| Description of model used (eg, decision tree, state transition, microsimulation) | × | |
| Modeling assumptions | × | × |
| Software used | × | |
| Identification of key outcomes | × | |
| Complete information on sources of effectiveness data, cost data, and preference weights | × | × |
| Methods for obtaining estimates of effectiveness (including approaches used for evidence synthesis) | × | × |
| Methods for obtaining estimates of costs and preference weights | × | × |



© 2026 Zhu L et al. JAMA Internal Medicine.

| | | |
|---|---|---|
| Critique of data quality | × | |
| Statement of costing year (ie, the year to which all costs have been adjusted for the analysis; eg, 2016) | × | |
| Statement of method used to adjust costs for inflation | × | |
| Statement of type of currency | × | |
| Source and methods for obtaining expert judgment if applicable | NA | |
| Statement of discount rates | × | |
| **Impact Inventory** | | |
| Full accounting of consequences within and outside the health care sector | NA[a] | |
| **Results** | | |
| Results of model validation | ×[b] | |
| Reference case results (discounted and undiscounted): total costs and effectiveness, incremental costs and effectiveness, incremental cost-effectiveness ratios, measures of uncertainty | × | × |
| Disaggregated results for important categories of costs, outcomes, or both | × | × |
| Results of sensitivity analysis | × | × |
| Other estimates of uncertainty | × | |
| Graphical representation of cost-effectiveness results | × | |
| Graphical representation of uncertainty analyses | × | × |
| Aggregate cost and effectiveness information | × | |
| Secondary analyses | × | |
| **Disclosures** | | |
| Statement of any potential conflicts of interest due to funding source, collaborations, or outside interests | × | |
| **Discussion** | | |
| Summary of reference case results | × | |
| Summary of sensitivity of results to assumptions and uncertainties in the analysis | × | |
| Discussion of the study results in the context of results of related cost-effective analyses | × | |
| Discussion of ethical implications (eg, distributive implications relating to age, disability, or other characteristics of the population) | × | |
| Limitations of the study | × | |
| Relevance of study results to specific policy questions or decisions | × | |

a. We adopted a healthcare perspective for our analysis to inform health policymaking within the healthcare sector.
b. Further details have been described in our previous publications.[1,2]


© 2026 Zhu L et al. JAMA Internal Medicine.

**eTable 2. Input parameters for simulations using the dynamic network model of people who inject drugs (PWID)**

| Parameters | Values | Reference |
|---|---|---|
| **Injection drug use dynamics** | | |
| Monthly injecting initiation rate[a] | 0.002 | calibration |
| Monthly injecting cessation rate[b] | 0.014 | 3 |
| Monthly injecting relapse rate[b] | 0.033 | 3 |
| Probability of permanent cessation of injecting[b] | 0.13 | 3 |
| **Network simulations**[c] | | |
| Initial number of current PWID | 1000 | 4 and assumption |
| Initial number of former PWID | 373 | SNAP and assumption |
| Baseline HCV antibody positivity (%) | 59 | 4, SNAP |
| Mean degree (in sparse network) | 1.43 | 4, SNAP |
| Mean degree (in dense network) | 3 | 4 and supplement eTable2 |
| Ratio of mean degree between HCV antibody (+) and (-) PWID | 1.73 | 4, SNAP |
| Proportion of population with no partners (isolates) (%) | 37 | 4, SNAP |
| HCV sero-discordant partnerships (%) | 29 | 4, SNAP |
| Transitivity (GWESP density[d]) | 0.28 | 4, SNAP |
| Average equipment-sharing partner duration (years)[e] | 3 | 5, SNAP, and assumption |
| **HCV infection and progression** | | |
| Monthly transmission probability in discordant partnerships | 0.031 | 4 (calibration) |
| Spontaneous clearance of acute infection (%) | 25 | 6 |
| Probability of HCV seroconversion after different period of time following acute infection[f] | | 7-9 |
| 1 month | 0.05 | |
| 2 months | 0.5 | |
| 3 months | 0.9 | |
| 4 months and after | 0.99 | |
| Monthly progression probability F0-F1 | 0.008877 | |
| Monthly progression probability F1-F2 | 0.00681 | |
| Monthly progression probability F2-F3 | 0.0097026 | 10 |
| Monthly progression probability F3-F4 | 0.0096201 | |
| Monthly progression probability F4-decomp | 0.0097026 | |
| **HCV testing and DAA treatment** | | |
| HCV antibody test specificity[g] | 0.99945 | 11 |
| HCV antibody test sensitivity[g] | 1 | 11 |
| HCV RNA test specificity[g] | 0.999985 | 11 |
| HCV RNA testing sensitivity[g] | 1 | 11 |
| RNA testing rate after treatment completion (%)[h] | 76 | 12 |
| Relative risk of reinfection after cure (compared to primary infection)[i] | 0.34 | 13-16 |
| **Effects of SSP and MOUD** | | |
| Relative reduction in transmission probability when engaged in SSP | 50% | 17 |
| Engagement duration for SSP | 12 months | 18 |
| Engagement duration for MOUD | 4 months | 19 |

a. Number of new PWID per month was calculated by multiplying this rate and initial population size (1000). Initiation rate was calibrated to keep population size of current PWID stable.



© 2026 Zhu L et al. JAMA Internal Medicine.

b. We used Kaplan-Meier estimators of time to cessation and relapse (given cessation) in the ALIVE study to estimate the three parameters (details in Technical Appendix).
c. We used separable temporal exponential random graph models operationalized using the Statnet package[20] in R (version 4.3.2; R Foundation for Statistical Computing, Vienna, Austria) to simulate the dynamic injection network.
d. Proportion of 2-stars (two nodes connected to a common node) that are closed (formed triangle), which describes the tendency of the phenomenon “friend’s friend is more likely to be a friend”.
e. Analysis from the SNAP data shows average duration of “acquaintance” between injection partners was 10 years; in the cited study, the reported median duration of “know each other” was 10 months, and duration of “injected with each other” was 4.5 months. We hence assumed an average duration of 3 years in the main analysis.
f. Antibodies to HCV typically become detectable within 20 to 150 days after infection (mean 60 days), [7] so we assumed 5% becoming detectable within one month after infection, and 50% within two months after infection. After 12 weeks, more than 90% of patients will have a positive HCV antibody test, [8] so we assumed 90% within three months after infection. In rare cases, there are individuals who do not develop antibody, and we assumed this percentage to be 1%. [9]
g Using mean value of the approved assays.
h. We used percentage of people who use drugs (PWUD) that had SVR data to approximate this parameter, the cited study reported 87 PWUD (its Table 1) among which 66 had SVR data (its Figure 1).
i. In the two cited meta-analyses, the rate of HCV reinfection after treatment was 6.2/100 person-years among people who recently injected drugs, and the gender-weighted rate of HCV primary infection among PWID was 18/100 person-years. The relative risk of reinfection after treatment was thus estimated to be 6.2/18=0.34. The third citation reported high rates of early HCV reinfection after treatment, and that 80% of people continued using drugs during treatment; and the fourth citation of meta-analysis reported that treatment reduced drug use but reported no change in sharing behavior. Based on these references, we used this same relative risk for reinfection during treatment.

**eTable 3. Cumulative intervention costs and incremental intervention costs for jails per person-year detained over the first 2 years of interventions**

| Strategy | Costs (jail & community, 2025 $)[a] | | | Incremental costs for jail (2025 $)[b] | | | |
|---|---|---|---|---|---|---|---|
| | test | treat | navigation | test | treat | navigation | total |
| no intervention | 173 | 630 | 0 | ref | ref | ref | ref |
| test | 1210 | 2024 | 0 | 1037 | 0 | 0 | 1037 |
| test + HCV navigation | 1204 | 3225 | 403 | 1032 | 0 | 403 | 1435 |
| test & treat | 1199 | 3465 | 0 | 1027 | 1441 | 0 | 2468 |
| test & treat + HCV navigation | 1194 | 4314 | 402 | 1022 | 1089 | 402 | 2513 |

a Cumulative costs of HCV testing, treatment, and navigation occurred in jail and community over the first 2 years of interventions for each strategy, divided by the cumulative person-years of jail population during the first 2 years.

b Incremental costs of HCV testing, treatment, and navigation occurred in jail for each strategy relative to the no intervention strategy, divided by the cumulative person-years of jail population during the first 2 years. These costs were not directly recorded from the model simulations but calculated from the cumulative costs in different strategies. E.g., incremental testing cost for jails under the "test" strategy was calculated as the testing cost in the "test" strategy minus the testing cost in the "no intervention" strategy; incremental treatment cost for jails under the "test & treat" strategy was calculated as the difference in treatment costs between the "test & treat" strategy and the "test" strategy, as the latter reflects background treatment costs in the community (under "no intervention") plus additional treatment costs to the community resulting from increased testing in jails, and the difference reflects incremental treatment costs to jails. Similar logic were applied to all the calculations.



© 2026 Zhu L et al. JAMA Internal Medicine.

**eTable 4. Contributions of different components to the total cumulative costs over 60 years among different jail-based intervention strategies**

| Strategy | Cumulative cost of different component (2025 US dollars) | | | | |
|---|---|---|---|---|---|
| | injection | liver | test&treat | navigation | SSP&MOUD |
| no intervention | 800716716 | 135826313 | 4078750 | 0 | 9592855 |
| test | 809179447 | 123736852 | 14489857 | 0 | 9594349 |
| test + HCV navigation | 815173941 | 114984977 | 17331220 | 2282386 | 9599864 |
| test & treat | 814604104 | 115157557 | 18104704 | 0 | 9605540 |
| test & treat + HCV navigation | 818413911 | 109820112 | 19237605 | 2281618 | 9606489 |

Abbreviations: SSP, syringe services program; MOUD, medications for opioid use disorder.

© 2026 Zhu L et al. JAMA Internal Medicine.

**eTable 5. Cumulative per-person costs, QALYs, and ICERs of jail-based HCV interventions over 60 years with health care costs related to injection drug use reduced by 50% relative to the base-case values**

| Strategy | Cost($) | QALY | Incremental Cost ($) | QALYs gained | ICER ($/QALY gained) |
|---|---|---|---|---|---|
| no intervention | 354403 | 13.44 | | | Comparator |
| test | 356050 | 13.70 | | | Dominated |
| test & treat | 354605 | 13.87 | | | Dominated |
| test + HCV navigation | 355646 | 13.88 | | | Dominated |
| test & treat + HCV navigation | 354594 | 13.99 | 191 | 0.56 | 343 |


© 2026 Zhu L et al. JAMA Internal Medicine.

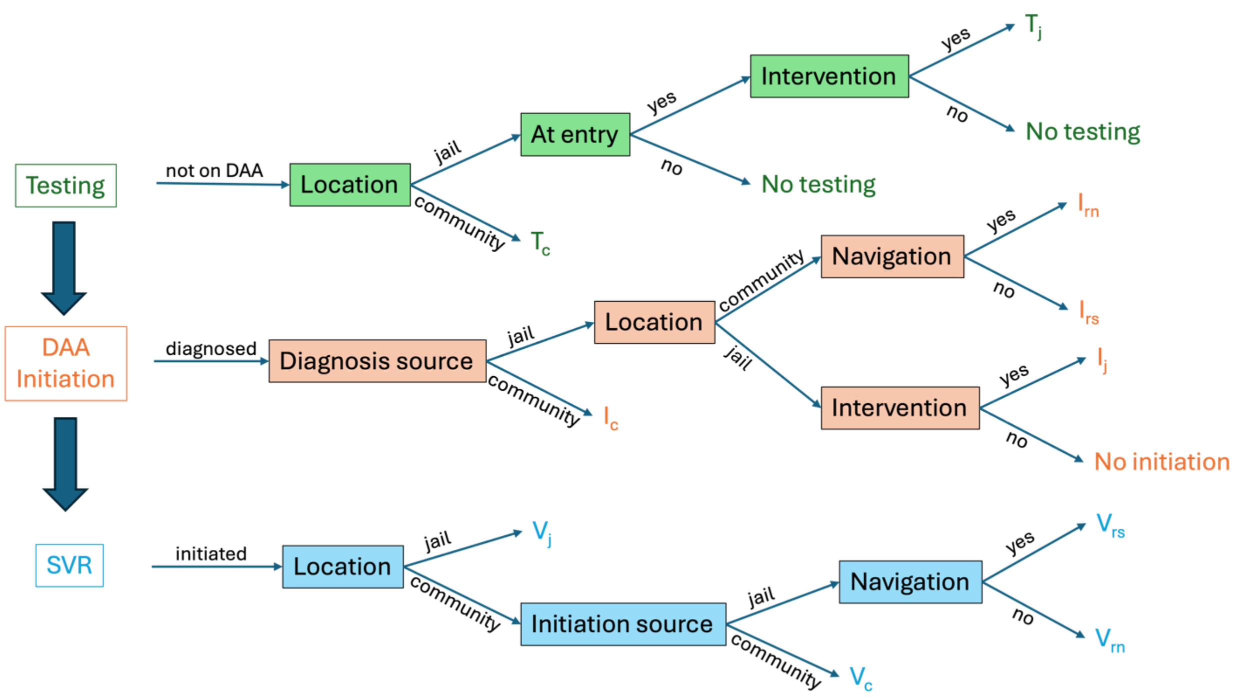


**eFigure 1. Decision trees of parameter selection on HCV testing, DAA initiation, and DAA completion**

The parameters used in the simulation for each individual depend on the current status and the intervention received. In these parameter symbols, letters of T, I, V represent testing, initiation, and sustained virologic response (SVR), respectively. Letters of j, r, and c represent jail, released, and community. Letters of s and n represent status quo and under navigation. The corresponding parameter names and values presented in **Table 1** are summarized below.

| Symbols | Parameter Names in Table 1 | Values |
|---|---|---|
| $T_c$ | % monthly HCV testing in community | 0.61 |
| $T_j$ | % tested at jail entry | 91 |
| $I_c$ | % DAA initiation among those diagnosed in community | 43 |
| $I_{rn}$ | % DAA initiation after release among those diagnosed in jail (under HCV navigation) | 47 |
| $I_{rs}$ | % DAA initiation after release among those diagnosed in jail (status quo) | 16 |
| $I_j$ | % DAA initiation in jail among those diagnosed in jail | 22 |
| $V_j$ | % SVR among those initiated in jail while detained in jail | 92 |
| $V_c$ | % SVR among those initiated in community | 90 |
| $V_{rs}$ | % SVR after release (status quo) among those initiated in jail | 67 |
| $V_{rn}$ | % SVR after release (under HCV navigation) among those initiated in jail | 75 |


© 2026 Zhu L et al. JAMA Internal Medicine.

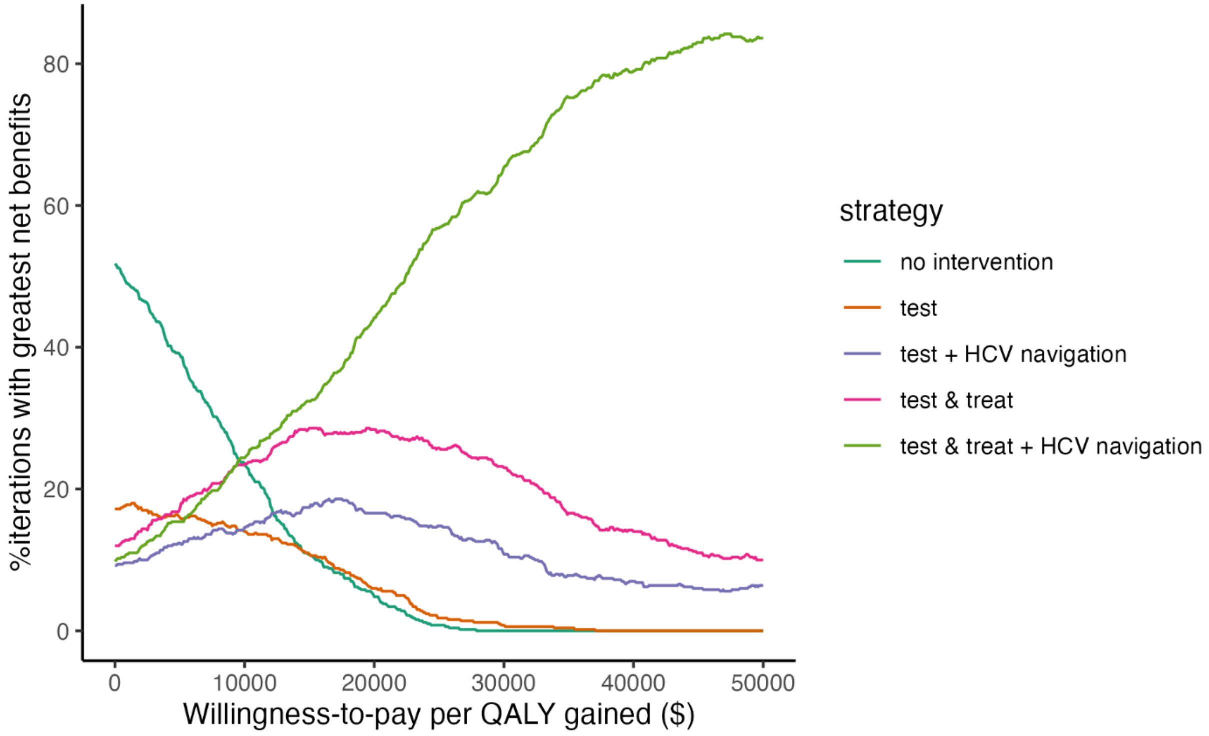


**eFigure 2. Stochastic uncertainty: percentage of simulation iterations in which each strategy had the greatest net benefits**

© 2026 Zhu L et al. JAMA Internal Medicine.

**eAppenidx. Technical Appendix**

## 1. Age distributions

### 1.1 Initial network and migrated individuals

We fitted normal, lognormal, gamma and Weibull distributions to the age pattern observed in the SNAP PWID sample and selected the lognormal distribution as the best fitting result based on AIC and BIC values.

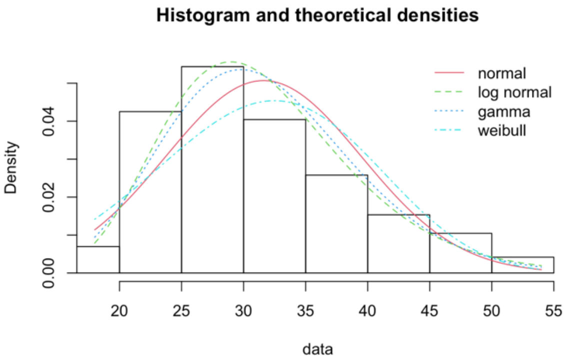


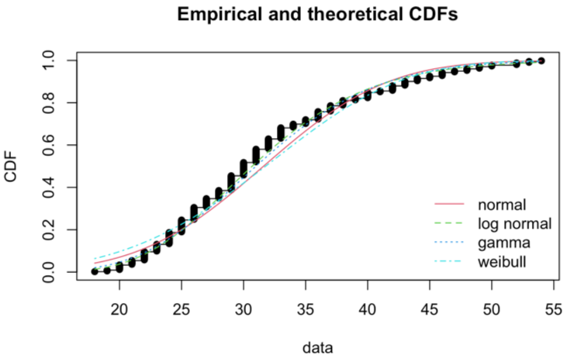


We used the fitted distribution (mean log=3.424, sd log=0.241) truncating at the minimum age of injection initiation = 13 to assign age distributions to the initial network and to individuals who migrate into the network.

### 1.2 New injectors

We identified the best-fitting distribution to the observed distribution of ages at injection initiation among the SNAP PWID following the same approach as above, with the lognormal distribution selected as above.

© 2026 Zhu L et al. *JAMA Internal Medicine*.

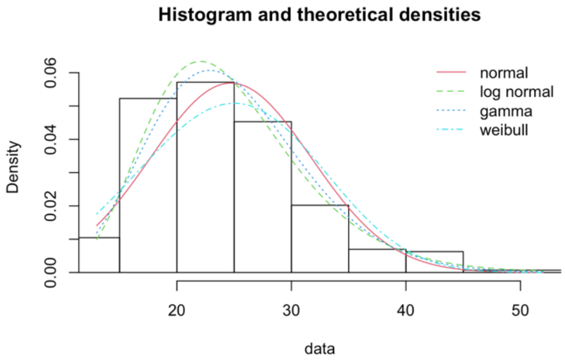


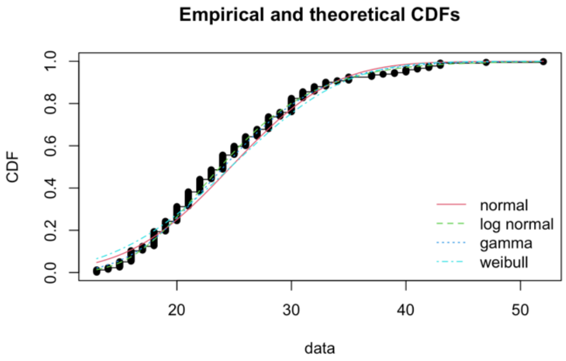


We used the fitted distribution (mean log=3.169, sd log=0.275) truncating at the minimum age of injection initiation = 13 to assign the age distribution for new injectors.

## 1.3 Aging

We updated age for all simulated individuals in the model at yearly intervals.

© 2026 Zhu L et al. *JAMA Internal Medicine*.

## 2. Fibrosis distribution

We assigned F0 to individuals who have never been infected. For individuals with current or former infection, we gathered numbers of patients in different fibrosis stages (none/mild, moderate, and advanced/cirrhosis) by birth cohort (born before 1945, 1945-1965, and after 1965) and HCV infection status (currently infected and resolved/cured) from Table 1 in the Klevens et al. study. [21] We converted the numbers of patients to percentages, and mapped none/mild to F0/F1, moderate to F1/F2, and advanced/cirrhosis to F3/F4/decompensated according to matching of FIB-4, Ishak 6, and METAVIR (FIB-4 maps to Ishak 0-1, 2-3, 4-6; Ishak 0-1 maps to METAVIR F0/F1; Ishak 2-3 maps to METAVIR F1/F2; and Ishak 4-6 maps to F3/F4). We implemented 50/50 splits between F0 and F1, F1 and F2, and 50/40/10 split between F3, F4, and decompensated. The table below shows results of these calculations.

Table Fibrosis distribution among individuals with current or former HCV infection

| Age | Infection Status | F0 | F1 | F2 | F3 | F4 | Decomp |
|---|---|---|---|---|---|---|---|
| 65-100 | Current | 0.0372 | 0.2665 | 0.2293 | 0.2335 | 0.1868 | 0.0467 |
| 65-100 | Resolved/Cured | 0.1423 | 0.4221 | 0.2798 | 0.0779 | 0.0623 | 0.0156 |
| 45-64 | Current | 0.1465 | 0.3641 | 0.2176 | 0.1359 | 0.1087 | 0.0272 |
| 45-64 | Resolved/Cured | 0.3303 | 0.4693 | 0.1390 | 0.0307 | 0.0245 | 0.0061 |
| 0-44 | Current | 0.3981 | 0.4740 | 0.0759 | 0.0260 | 0.0208 | 0.0052 |
| 0-44 | Resolved/Cured | 0.4713 | 0.4946 | 0.0233 | 0.0054 | 0.0043 | 0.0011 |

© 2026 Zhu L et al. *JAMA Internal Medicine*.

## 3. Initiation, cessation, and relapse of injection drug use

### 3.1 Initiation

We calibrated the initiation rate to make the size of the active injection network stable (around 1000 current PWID) in the status quo (no intervention) scenario. We assumed that each person is HCV naïve at injection drug use initiation.[22]

### 3.2 Cessation and relapse

We estimated monthly rates of injecting cessation and injecting relapse, and the probability of permanent cessation, from the ALIVE cohort study conducted in Baltimore, MD over 1988 to 2000. [3] The following steps were used:

a. We digitized the time to cessation and relapse curves reported by Shah NG et al. [3] in Figure 1 (Kaplan–Meier of observed time from baseline to first cessation of injection, and time to first relapse to injection following cessation), extracting 25 data points from each curve.
b. We the asymptote of the relapse curve to estimate the probability of permanent cessation, which was approximately 13%.
c. We fitted an exponential function to the digitized data points of the cessation curve with a forced intercept of 100. The exponential function was estimated as Y = 100 exp (-0.168 X), with an adjusted R-squared of 0.9988. Based on the estimated coefficient, we computed the monthly cessation rate to be 0.168/12 = 0.014.
d. For the relapse curve, we first converted the Y values to be conditional on ever relapsing (i.e., allowing for the curve to approach an asymptote at 13%): Z = (Y-13)/(100-13)×100. Then we fitted an exponential function using the same method as the cessation curve, and the result was Z = 100 exp (-0.454 X), with an adjusted R-squared of 0.9589. We computed the monthly relapse rate as 0.454/100×(100-13)/12 = 0.033.

PWID who cease injection are characterized as "former PWID" and pause their injection partnerships. When former PWID relapse, they return to the active PWID network and re-acquire previous partners if they remain in the network. PWID who initiate injection are assigned partnerships to preserve network mean degree; partnerships of PWID who die are removed permanently.

© 2026 Zhu L et al. *JAMA Internal Medicine*.

## 4. Mortality

### 4.1 Age dependent base mortality

We used 2019 United States life tables.[23] We downloaded life tables for males (https://ftp.cdc.gov/pub/Health_Statistics/NCHS/Publications/NVSR/70-19/Table02.xlsx) and females (https://ftp.cdc.gov/pub/Health_Statistics/NCHS/Publications/NVSR/70-19/Table02.xlsx) on May 1 2023. We converted mortality probabilities to rates, calculated both-sexes rates using the sex distribution reported for the SNAP PWID sample (59% male), and converted yearly rates back to monthly probabilities. The probability of death at age 100 was set to 1.

### 4.2 Age-dependent drug-use-related standardized mortality ratio (SMR)

We identified SMRs associated with drug use from the Evans et al.[24] cohort study among individuals accessing pharmacological treatment for opioid dependence, which reported point estimates of 1.8 and 6.1 for SMRs among former and current drug use, respectively, and an overall estimate of 4.5. The study also showed that the SMR decreased by age (Table 3 in the publication), consistent with results of a systematic review [25] and analysis of the ALIVE study. [26] Based on these findings, we estimated age-dependent drug-use-related SMRs using the following calculations.

We calculated the ratios of current or former drug use SMRs to SMRs in the overall cohort with an offset of 1:

$$\text{ratio}_{\text{current}} = \frac{\text{current SMR} - 1}{\text{overall SMR} - 1} = \frac{6.1 - 1}{4.5 - 1} = 1.457$$

$$\text{ratio}_{\text{former}} = \frac{\text{former SMR} - 1}{\text{overall SMR} - 1} = \frac{1.8 - 1}{4.5 - 1} = 0.229$$

We fitted a regression to reported age-specific SMRs in Evans et al., which resulted in the estimated function $\log(\text{SMR} - 1) = 2.6193 - 0.0304 \times \text{Age}$. We used this estimated regression to generate overall SMRs by age, and then used the two ratios calculated in the first step to estimate SMRs by age for current and former injectors.

### 4.3 Excess mortality due to F4 and decompensation

We calculated excess mortality for compensated and decompensated cirrhosis from results reported in a cohort study by Bruno et al.[27] as follows:

a. From numbers in Figure 1 in the report, the number of liver deaths and annual rate among untreated patients were 60 and 3/100 person-years (py), respectively, implying total person-time of 60/0.03=2000 py; the reported number of liver deaths and annual rate among treated patients without sustained virological response (SVR) were 46 and 2.4/100 py, respectively, implying total person-time is 46/0.024=1917 py.

© 2026 Zhu L et al. *JAMA Internal Medicine*.

b. We combined decompensation and HCC in our model as one group representing severe liver disease, so our calculation from this study was also based on this grouping.
c. From Figure 3 in the report, the number of deaths with all combinations of decompensation and HCC from untreated and treated patients without SVR was 30+10+15+47=102, and total person-years was 301+31+251+80=663.
d. Combining numbers in steps a and c, the number of deaths for F4 was 60+46-102=4, person-time was 2000+1917-663=3254, and the annual rate was 4/3254=0.12/100 py, implying a monthly excess mortality rate due to F4 of 0.012/100/12=0.0001.
e. Similarly, the number of deaths for decompensated cirrhosis (plus HCC) was 102/663=15.38/100 py, implying a monthly excess mortality rate due to decompensation (plus HCC) of 0.0128.

## 4.4 Impact of sustained virologic response (SVR) on excess mortality due to F4 or decompensation

Based on results of an international, multicenter, long-term follow-up study by van der Meer et al. (Table 4 in the report), [28] we multiplied the hazard ratio (HR) for liver-related mortality by SVR and Fibrosis Ishak 6 (corresponding to F4, and we assume similar HR for decompensation) 0.06×4.84=0.29 to derive the HR for liver-related mortality following SVR among F4 and decompensation patients.

© 2026 Zhu L et al. *JAMA Internal Medicine*.

## 5. Background health utilities

We used age-specific background health utilities based on population norms measured with the EQ-5D[29] for each individual.

Health utilities for ages below 20 years or ages 90 and older were not reported in the previous study. We set utilities in the 14-19-year age group equal to those in the age group 20-29; and those of age above 89 equal to those reported for ages 80-89.

© 2026 Zhu L et al. *JAMA Internal Medicine*.

## 6. Health utilities related to liver conditions and HCV infection

We used values reported in a systematic review and meta-analysis[30] to determine model inputs for utilities related to liver disease and HCV infection status. Calculations are summarized below.

a. The health utility for each individual is determined by age, injection status, and HCV/liver disease status, assuming that each of these three dimensions is independent, and operationalized using a multiplicative model.
b. Utilities for states relating to liver disease were normalized in relation to the utility for sustained virologic response (SVR) to HCV treatment, which we assigned a dimension-specific value of 1.0. We computed utility values for each other state in this dimension as the ratio of the estimated value for that state to the estimated value for SVR, as follows:

   F0-F3 (mild to moderate fibrosis): 0.751/0.786 = 0.96

   F4 (compensated cirrhosis): 0.671/0.786 = 0.85

   Decompensated cirrhosis: 0.602/0.786 = 0.77

c. For changes in utility following treatment, we assumed that those treated in starting states F0-F3 and experiencing SVR would move to a utility value of 1.0, i.e. assumed that effective treatment would reverse disutilities associated with untreated mild to moderate fibrosis. For those treated in starting states of F4 or decompensated cirrhosis, we assumed that SVR would reduce liver disease disutility by a factor of 0.29, which we chose to be the same as the assumed reduction in excess mortality due to F4 and decompensated (as described above, in section 3.4). Based on these assumptions, we calculated utilities for F4 and decompensated after SVR as follows:

   F4: $1 - (1 - 0.85) \times 0.29 = 0.96$

   Decompensated: $1 - (1 - 0.77) \times 0.29 = 0.93$

© 2026 Zhu L et al. *JAMA Internal Medicine*.

## 7. Healthcare costs associated with injection drug use

We estimated other healthcare costs (excluding costs of MOUD, detox, etc.) associated with injection drug use and attached them to each individual depending on their age and drug use status. We estimated the costs from the NIDA Clinical Trials Network CTN-0051: Extended-Release Naltrexone vs. Buprenorphine for Opioid Treatment (X:BOT) trial, a multisite, 2-arm, open-label, randomized controlled trial that tested the effectiveness of XR-NTX versus BUP-NX with regard to patient survival, free of opioid relapse, at 24 weeks. [31-33] We contacted the authors (Sean Murphy and Danielle Ryan) to obtain healthcare utilization costs stratified by age, injection drug use status, and treatment. The original results were reported as costs over 6 months and in 2016 US dollars. We converted them into monthly costs in 2025 dollars using medical care Consumer Price Index (CPI). We presented mean costs across all age groups in Table 1 in the manuscript. The table below shows the detailed age stratified costs that we used in our simulation.

Table Healthcare utilization costs associated with injection drug use

| injection status | age group | cost (6 months, 2016 $) | monthly | 2025 $ |
|---|---|---|---|---|
| current | <24 years of age | 8731 | 1455 | 1814 |
| | 25-44 | 12174 | 2029 | 2529 |
| | 45-99 | 10363 | 1727 | 2153 |
| former (not on MOUD) | <24 years of age | 6380 | 1063 | 1325 |
| | 25-44 | 9174 | 1529 | 1906 |
| | 45-99 | 7522 | 1254 | 1563 |
| former (on MOUD) | <24 years of age | 4587 | 765 | 953 |
| | 25-44 | 6599 | 1100 | 1371 |
| | 45-99 | 5498 | 916 | 1142 |

© 2026 Zhu L et al. *JAMA Internal Medicine*.

## 8. Healthcare costs related to HCV and liver conditions

We used costs reported in a retrospective, matched cohort study with a large claims database [34] to calculate background healthcare costs related to HCV and liver conditions. In Table 4 in the cited paper, which summarized the incremental all-cause per patient per year healthcare costs for patients with HCV relative to matched comparison group, we obtained sample sizes, mean inpatient costs, pharmacy costs, and total costs for four HCV and liver condition groups including HCV without liver disease, compensated cirrhosis, decompensated cirrhosis, and HCC. We then adjusted the inpatient costs with a cost-to-charge ratio of 0.329, [35] and subtracted pharmacy costs from total costs to obtain the final background healthcare costs for the four HCV and liver condition groups. To avoid potential measurement error relating to small samples, we collapsed the four categories into two categories, combining HCV without liver disease and compensated cirrhosis into a group spanning F0-F4, and combining decompensated cirrhosis and HCC into a "severe liver disease" category. For each combined category we computed costs as a sample-weighted average of the component category costs. We adjusted costs to 2025 US dollars using medical care CPI. For patients without HCV or cured, we multiplied costs by a factor  calculated from total monthly HCV-related healthcare costs, stratified by SVR attainment status, reported in Table 4 in a matched study from US claims database, [36] which equals 717/1436=0.5. We divided these costs by 12 to obtain monthly costs as our input. The calculations are summarized in the table below.

| costs | HCV without liver disease (F0-F3) | Compensated cirrhosis (F4) | Decompensated cirrhosis | HCC |
|---|---|---|---|---|
| N | 26977 | 1521 | 4249 | 959 |
| all-cause healthcare cost total | 5870 | 5330 | 27845 | 43671 |
| in-patient cost | 810 | 974 | 15464 | 17197 |
| adjusted in-patient cost | 266 | 320 | 5088 | 5658 |
| all-cause, adjusted in-patient | 5326 | 4676 | 17469 | 32132 |
| pharmacy | 2659 | 3102 | 0 | 0 |
| all-case, adjusted in-patient, excluding pharmacy | 2667 | 1574 | 17469 | 32132 |
| adjusted to 2025 | 4105 | 2423 | 26881 | 49444 |
| | weighted F0-F4 | 4015 | weighted DC/HCC | 31036 |
| | non-HCV | 2007 | | 15518 |

Monthly costs input (20205 $):

F0-F4 with HCV: 335

DC with HCV: 2586

© 2026 Zhu L et al. *JAMA Internal Medicine*.

F0-F4 without HCV: 167

DC without HCV: 1293

© 2026 Zhu L et al. *JAMA Internal Medicine*.

## 9. Weighted population size

We computed the cumulative per-person costs and QALYs by dividing the total cumulative costs and QALYs of the simulated PWID population over 60 years by a weighted cohort size estimate, which accounted for individuals entering the cohort at different times ($N_w = \sum_{t=0}^{120} N_t \times \frac{120-t}{120}$, with t representing time in months, and $N_t$ representing the number of individuals entering the cohort at month t).

© 2026 Zhu L et al. *JAMA Internal Medicine*.

## References

1. Zhu L, Havens JR, Rudolph AE, et al. Hepatitis C virus transmission among people who inject drugs in rural United States: mathematical modeling study using stochastic agent-based network simulation. *American Journal of Epidemiology* 2025: kwaf052.

2. Zhu L, Furukawa NW, Thompson WW, et al. Health and Economic Impact of Periodic Hepatitis C Virus Testing Among People Who Inject Drugs. JAMA Health Forum; 2025: American Medical Association; 2025. p. e251870-e.

3. Shah NG, Galai N, Celentano DD, Vlahov D, Strathdee SA. Longitudinal predictors of injection cessation and subsequent relapse among a cohort of injection drug users in Baltimore, MD, 1988–2000. *Drug and alcohol dependence* 2006; **83**(2): 147-56.

4. Zhu L, Thompson WW, Hagan L, et al. Potential impact of curative and preventive interventions toward hepatitis C elimination in people who inject drugs–A network modeling study. *International Journal of Drug Policy* 2024; **130**: 104539.

5. Morris MD, Evans J, Montgomery M, et al. Intimate injection partnerships are at elevated risk of high-risk injecting: a multi-level longitudinal study of HCV-serodiscordant injection partnerships in San Francisco, CA. *PloS one* 2014; **9**(10): e109282.

6. Smith DJ, Jordan AE, Frank M, Hagan H. Spontaneous viral clearance of hepatitis C virus (HCV) infection among people who inject drugs (PWID) and HIV-positive men who have sex with men (HIV+ MSM): a systematic review and meta-analysis. *BMC infectious diseases* 2016; **16**(1): 1-13.

7. Busch MP, Page Shafer KA. Acute-phase hepatitis C virus infection: implications for research, diagnosis, and treatment. The University of Chicago Press; 2005. p. 959-61.

8. Rena K. Fox MAC. Diagnosis of Acute HCV Infection. 2021. https://www.hepatitisc.uw.edu/go/screening-diagnosis/acute-diagnosis/core-concept/all.

© 2026 Zhu L et al. *JAMA Internal Medicine*.

9. Stramer SL, Glynn SA, Kleinman SH, et al. Detection of HIV-1 and HCV infections among antibody-negative blood donors by nucleic acid–amplification testing. *New England Journal of Medicine* 2004; **351**(8): 760-8.
10. Erman A, Krahn MD, Hansen T, et al. Estimation of fibrosis progression rates for chronic hepatitis C: a systematic review and meta-analysis update. *BMJ open* 2019; **9**(11): e027491.
11. FDA. Complete List of Donor Screening Assays for Infectious Agents and HIV Diagnostic Assays. 2023. https://www.fda.gov/vaccines-blood-biologics/complete-list-donor-screening-assays-infectious-agents-and-hiv-diagnostic-assays#Anti-HCV%20Assays%20(detect%20antibodies%20to%20Hepatitis%20C%20Virus%20Encoded%20Antigen).
12. Cornberg M, Stoehr A, Naumann U, et al. Real-World Safety, Effectiveness, and Patient-Reported Outcomes in Patients with Chronic Hepatitis C Virus Infection Treated with Glecaprevir/Pibrentasvir: Updated Data from the German Hepatitis C-Registry (DHC-R). *Viruses* 2022; **14**(7): 1541.
13. Hajarizadeh B, Cunningham EB, Valerio H, et al. Hepatitis C reinfection after successful antiviral treatment among people who inject drugs: A meta-analysis. *Journal of hepatology* 2020; **72**(4): 643-57.
14. Esmaeili A, Mirzazadeh A, Carter GM, et al. Higher incidence of HCV in females compared to males who inject drugs: a systematic review and meta-analysis. *Journal of viral hepatitis* 2017; **24**(2): 117-27.
15. Valencia J, Alvaro-Meca A, Troya J, et al. High rates of early HCV reinfection after DAA treatment in people with recent drug use attended at mobile harm reduction units. *International Journal of Drug Policy* 2019; **72**: 181-8.
16. Caven M, Malaguti A, Robinson E, Fletcher E, Dillon JF. Impact of hepatitis C treatment on behavioural change in relation to drug use in people who inject drugs: a systematic review. *International Journal of Drug Policy* 2019; **72**: 169-76.
17. Platt L, Minozzi S, Reed J, et al. Needle syringe programmes and opioid substitution therapy for preventing hepatitis C transmission in people who inject drugs. *Cochrane Database of Systematic Reviews* 2017; (9).

© 2026 Zhu L et al. *JAMA Internal Medicine*.

18. Gindi RM, Rucker MG, Serio-Chapman CE, Sherman SG. Utilization patterns and correlates of retention among clients of the needle exchange program in Baltimore, Maryland. *Drug and alcohol dependence* 2009; **103**(3): 93-8.
19. Krawczyk N, Williams AR, Saloner B, Cerdá M. Who stays in medication treatment for opioid use disorder? A national study of outpatient specialty treatment settings. *Journal of Substance Abuse Treatment* 2021; **126**: 108329.
20. Statnet Development Team (Pavel N. Krivitsky MSH, David R. Hunter, Carter T. Butts, Chad Klumb, Steven M. Goodreau, and Martina Morris) statnet: Software tools for the Statistical Modeling of Network Data. . (2003-2020). http://statnet.org.
21. Klevens RM, Canary L, Huang X, et al. The burden of hepatitis C infection–related liver fibrosis in the United States. *Clinical Infectious Diseases* 2016; **63**(8): 1049-55.
22. Fuller CM, Ompad DC, Galea S, Wu Y, Koblin B, Vlahov D. Hepatitis C incidence—a comparison between injection and noninjection drug users in New York City. *Journal of Urban Health* 2004; **81**(1): 20-4.
23. Arias E, Xu J, Tejada-Vera B, Bastian B. United States life tables, 2019. 2022.
24. Evans E, Li L, Min J, et al. Mortality among individuals accessing pharmacological treatment for opioid dependence in California, 2006–10. *Addiction* 2015; **110**(6): 996-1005.
25. Larney S, Tran LT, Leung J, et al. All-cause and cause-specific mortality among people using extramedical opioids: a systematic review and meta-analysis. *JAMA psychiatry* 2020; **77**(5): 493-502.
26. Cepeda JA, Astemborski J, Kirk GD, Celentano DD, Thomas DL, Mehta SH. Rising role of prescription drugs as a portal to injection drug use and associated mortality in Baltimore, Maryland. *PLoS One* 2019; **14**(3): e0213357.
27. Bruno S, Zuin M, Crosignani A, et al. Predicting Mortality Risk in Patients With Compensated HCV-Induced Cirrhosis: A Long-Term Prospective Study. *Official journal of the American College of Gastroenterology| ACG* 2009; **104**(5): 1147-58.

© 2026 Zhu L et al. *JAMA Internal Medicine*.

28. van der Meer AJ, Veldt BJ, Feld JJ, et al. Association between sustained virological response and all-cause mortality among patients with chronic hepatitis C and advanced hepatic fibrosis. *Jama* 2012; **308**(24): 2584-93.
29. Hanmer J, Lawrence WF, Anderson JP, Kaplan RM, Fryback DG. Report of nationally representative values for the noninstitutionalized US adult population for 7 health-related quality-of-life scores. *Medical Decision Making* 2006; **26**(4): 391-400.
30. Saeed YA, Phoon A, Bielecki JM, et al. A systematic review and meta-analysis of health utilities in patients with chronic hepatitis C. *Value in Health* 2020; **23**(1): 127-37.
31. Lee JD, Nunes EV, Bailey GL, et al. NIDA Clinical Trials Network CTN-0051, extended-release naltrexone vs. buprenorphine for opioid treatment (X: BOT): study design and rationale. *Contemporary clinical trials* 2016; **50**: 253-64.
32. McCollister KE, Leff JA, Yang X, et al. Cost of pharmacotherapy for opioid use disorders following inpatient detoxification. *The American journal of managed care* 2018; **24**(11): 526.
33. Murphy SM, McCollister KE, Leff JA, et al. Cost-effectiveness of buprenorphine–naloxone versus extended-release naltrexone to prevent opioid relapse. *Annals of internal medicine* 2019; **170**(2): 90-8.
34. McAdam-Marx C, McGarry LJ, Hane CA, Biskupiak J, Deniz B, Brixner DI. All-cause and incremental per patient per year cost associated with chronic hepatitis C virus and associated liver complications in the United States: a managed care perspective. *Journal of Managed Care Pharmacy* 2011; **17**(7): 531-46.
35. Chhatwal J, Ferrante SA, Brass C, et al. Cost-effectiveness of boceprevir in patients previously treated for chronic hepatitis C genotype 1 infection in the United States. *Value in Health* 2013; **16**(6): 973-86.
36. Davis KL, Mitra D, Medjedovic J, Beam C, Rustgi V. Direct economic burden of chronic hepatitis C virus in a United States managed care population. *Journal of clinical gastroenterology* 2011; **45**(2): e17-e24.

© 2026 Zhu L et al. *JAMA Internal Medicine*.
