# Supplementary material for: Health Benefits, Costs, and Cost-Effectiveness of Jail-Based Hepatitis C Elimination Strategies
Source: JAMA Intern Med. 2026 Mar 23;186(5):585–95. doi: 10.1001/jamainternmed.2026.0190 (PMC13010197; doi:10.1001/jamainternmed.2026.0190)
Supplement: Supplement 2. — Data Sharing Statement [file jamainternmed-e260190-s002.pdf]

Data Sharing Statement

Zhu. Health Benefits, Costs, and Cost-Effectiveness of Jail-Based Hepatitis C Elimination Strategies. *JAMA Intern Med*. Published March 23, 2026.
doi:10.1001/jamainternmed.2026.0190

Data
Data available: No

Additional Information
Explanation for why data not available: No data were collected in this study. We used published data to inform model parameterization.
